# Chimeric Antigen Receptor‐Macrophage Therapy Enters the Clinic: The First‐in‐Human Trial for HER2^+^ Solid Tumors

**DOI:** 10.1002/mco2.70374

**Published:** 2025-08-27

**Authors:** Wenxue Ma, Catriona Jamieson

**Affiliations:** ^1^ Department of Medicine Sanford Stem Cell Institute Moores Cancer Center University of California San Diego La Jolla California USA; ^2^ CIRM Alpha Stem Cell Clinic University of California San Diego La Jolla California USA

1

In a recent study published in Nature Medicine (2025), Reiss et al. [1] reported the first‐in‐human Phase 1 trial of CT‐0508, a chimeric antigen receptor macrophage (CAR‐M) therapy targeting HER2‐overexpressing solid tumors. This trial demonstrated safety, feasibility, and early immune activity in heavily pretreated patients, marking the first clinical validation of macrophage‐based immunotherapy. This milestone highlights the potential of engineered macrophages to overcome tumor microenvironment (TME)‐mediated resistance, signaling a paradigm shift for treating solid tumors refractory to current therapies.

Fourteen patients with HER2‐overexpressing tumors, including breast, gastroesophageal, and salivary duct carcinomas, were enrolled and received CT‐0508 without prior lympho‐depleting chemotherapy. Two dosing regimens were explored: fractionated (Group 1) and bolus (Group 2). CAR‐Ms were generated from autologous monocytes using a replication‐incompetent adenoviral vector (Ad5.F35), achieving high viability, purity, and CAR expression across all manufactured products. Preclinical assays demonstrated HER2‐specific cytotoxicity, phagocytosis, and secretion of proinflammatory cytokines upon antigen engagement.

Clinically, CT‐0508 exhibited a favorable safety profile. No dose‐limiting toxicities, Grade ≥3 cytokine release syndrome (CRS), or immune effector cell‐associated neurotoxicity syndrome (ICANS) were observed. Grade 1–2 CRS occurred in nine patients and was managed without corticosteroids or intensive supportive care. Importantly, the trial avoided the use of lympho‐depleting regimens such as cyclophosphamide or fludarabine, which are standard in CAR‐T therapies, thereby supporting a more tolerable, outpatient‐based delivery model. This distinction represents a major advantage in the solid tumor space, where toxicity concerns and logistical burdens have limited broader access to cell therapy.

Although no objective responses (per RECIST v1.1) were observed, four of nine patients (44.5%) with HER2 immunohistochemistry (IHC) 3^+^ tumors achieved stable disease (SD), while all five patients with HER2 IHC 2^+^ tumors exhibited progressive disease (PD). Tumor volume reductions were noted in 41% of measurable lesions, and circulating tumor DNA (ctDNA) levels decreased in 62% of patients, including all those with SD. These molecular and radiographic evidence indicate biological activity, although transient. Interestingly, some patients exhibited ctDNA rebound at later timepoints, raising questions about the durability of response and the potential need for maintenance dosing or sequential therapeutic strategies. These observations reinforce the value of integrating longitudinal molecular monitoring in early‐phase trials to better understand the kinetics of response and identify early markers of relapse. A graphical overview summarizing the clinical workflow, immunologic changes, and future directions of CAR‐M therapy is provided in Figure 1.

**FIGURE 1:**
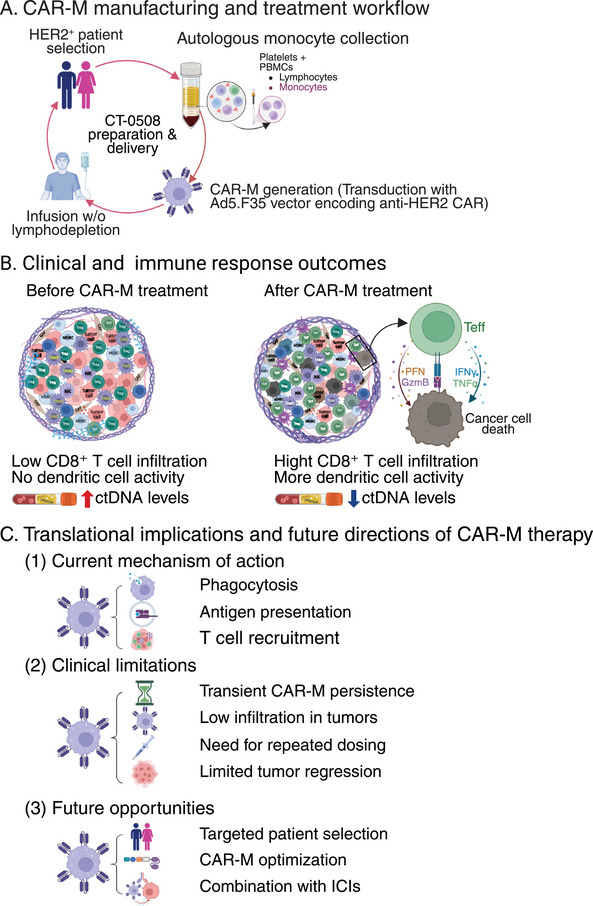
Clinical trial workflow, immune outcomes, and translational implications of chimeric antigen receptor macrophage (CAR‐M) therapy in HER2⁺ solid tumors. (A) CT‐0508, an autologous anti‐HER2 CAR‐M therapy, was manufactured from peripheral monocytes collected from HER2⁺ patients and transduced ex vivo with an Ad5.F35 vector encoding the anti‐HER2 CAR. Cells were infused without lympho‐depletion in a first‐in‐human clinical trial. (B) The tumor microenvironment (TME) before CAR‐M therapy was characterized by low CD8⁺ T‐cell infiltration, minimal dendritic cell (DC) activity, and elevated circulating tumor DNA (ctDNA) levels. Post‐treatment, the TME shows increased infiltration of CD8⁺ T cells and DCs, enhanced cytotoxic activity (granzyme B [GzmB], perforin [PFN], interferon gamma [IFNγ], tumor necrosis factor alpha [TNFα]), and reduced ctDNA levels. (C) Translational framework outlining CAR‐M mechanism of action (phagocytosis, antigen presentation, T‐cell recruitment), alongside current clinical limitations (transient persistence, low tumor infiltration, need for repeated dosing, limited tumor regression), and future opportunities including biomarker‐guided patient selection, CAR‐M optimization, and combination with immune checkpoint inhibitors (ICIs).

A striking feature of the study is the clear dependence of clinical benefit on HER2 expression intensity. Responses were exclusively observed in patients with HER2 IHC 3^+^ tumors, characterized by strong membranous staining. Non‐clinical benefit was observed among HER2 IHC 2^+^ patients. Furthermore, more than half of screening failures were attributed to HER2 downregulation at the time of enrollment, highlighting the plasticity of HER2 expression and its susceptibility to prior treatment pressure or tumor evolution [1]. These findings highlight the critical need for rigorous and real‐time biomarker evaluation to ensure optimal patient selection. As target expression heterogeneity continues to challenge the field, developing more dynamic patient stratification models, including serial biopsies or advanced imaging modalities, will be essential for identifying those most likely to benefit from CAR‐M therapy.

Mechanistically, CT‐0508 induced significant remodeling of the TME. Single‐cell RNA sequencing (scRNA‐seq) analysis and paired biopsies revealed increased antigen presentation gene signatures and enhanced infiltration of CD8^+^ T cells, many of which displayed clonotypic expansion and cytotoxic markers (granzyme B, perforin 1, IFN‐γ). These features suggest antigen spreading and reactivation of endogenous T‐cell responses. Patients achieving SD also showed higher baseline CD4:CD8 ratios and lower systemic inflammation, pointing to possible predictive markers for immunologic responsiveness. Importantly, these immune signatures resemble those seen in checkpoint blockade and suggest that CAR‐M may prime the TME for combinatorial strategies.

Despite these promising mechanistic insights, the therapeutic effects were transient. Most patients experienced disease progression within a few months. Limited CAR‐M persistence in circulation and low infiltration levels within tumor tissues, as assessed by RNAScope in situ RNA technology, likely contributed to this outcome. This raises critical questions about how to enhance CAR‐M retention, tumor homing, and intratumoral survival. Future approaches may include genetic modifications to enhance lifespan, co‐expression of chemokines or homing receptors, and integration with depot‐based delivery systems or repeated dosing strategies.

Importantly, this trial demonstrates that macrophages, historically difficult to transduce, can be successfully engineered using adenoviral platforms without compromising their phenotype or function. This technical advance overcomes a major bottleneck and paves the way for future iterations of CAR‐M with enhanced payloads, such as cytokine release constructs, antigen‐presenting modules, or immune checkpoint regulators [2]. Additionally, the manufacturing process, performed without ex vivo expansion, enhances the feasibility and scalability of autologous CAR‐M therapy for broader clinical application.

Beyond HER2 targeting, the broader implications of this trial lie in its validation of macrophages as a viable immune effector cell type for adoptive immunotherapy. Unlike T cells, macrophages are innate immune cells capable of infiltrating tumor stroma, performing phagocytosis, and activating adaptive immunity through antigen presentation. Their myeloid lineage reduces susceptibility to exhaustion and supports potential for off‐the‐shelf, allogeneic applications. As engineering platforms evolve, CAR‐Ms may be tailored to address multiple tumor antigens, modulate polarization states, or carry synthetic payloads to reprogram the TME.

From a translational perspective, this trial highlights several critical considerations for advancing CAR‐M platforms. These include (i) optimizing patient selection based on both tumor antigen density and immune contexture; (ii) integrating real‐time, multiomic monitoring to guide therapeutic decisions; and (iii) rationally designing combination regimens, particularly with ICIs, toll‐like receptor (TLR) agonists, or metabolic modulators to enhance efficacy and overcome resistance. Collaborative efforts between academic centers and industry partners will be essential to accelerate innovation and clinical translation.

Moving forward, enhancing the persistence and expansion of CAR‐Ms, possibly through cytokine support, synthetic constructs, or combination with immune checkpoint blockade, may improve clinical outcomes [3]. Biomarker‐guided patient stratification, especially based on HER2 IHC scores and immune fitness signatures, will be critical in optimizing therapeutic outcomes. Furthermore, strategies to convert early immune activation into sustained tumor regression, such as in vivo reprogramming, combination immunotherapies, or CAR‐M modifications for improved antigen retention, are essential for long‐term efficacy.

In summary, this first‐in‐human trial of CT‐0508 establishes a clinical proof‐of‐concept for CAR‐M therapy in HER2‐positive solid tumors. While limitations remain, the study demonstrates that macrophage‐based therapies can be safely administered, remodel the TME, and engage adaptive immunity. CAR‐Ms represent a promising new frontier in cancer immunotherapy, offering a unique mechanism of action that may complement existing T‐cell‐based approaches [4].

## Author Contributions

Wenxue Ma: conceptualization, investigation, writing—original draft, validation, visualization, writing—review and editing, software, formal analysis, project administration, data curation, supervision, resources. Catriona Jamieson: conceptualization, funding acquisition, writing—review and editing, project administration, supervision, resources, validation. Both authors have read and approved the final manuscript.

## Ethics Statement

The authors have nothing to report.

## Conflicts of Interest

The authors declare no conflicts of interest.

## Data Availability

Data availability is not applicable to this highlight as no new data were created or analyzed in it.
